# Interactive Teleoperation of an Articulated Robotic Arm Using Vision-Based Human Hand Tracking

**DOI:** 10.3390/biomimetics11020151

**Published:** 2026-02-19

**Authors:** Marius-Valentin Drăgoi, Aurel-Viorel Frimu, Andrei Postelnicu, Roxana-Adriana Puiu, Gabriel Petrea, Alexandru Hank

**Affiliations:** 1 Faculty of Industrial Engineering and Robotics, National University of Science and Technology POLITEHNICA Bucharest, 060042 Bucharest, Romania; andrei.postelnicu04@stud.fiir.upb.ro (A.P.); roxana.puiu@upb.ro (R.-A.P.); gabriel.petrea@upb.ro (G.P.); 2 Faculty of Engineering in Foreign Languages, National University of Science and Technology POLITEHNICA Bucharest, 060042 Bucharest, Romania; aurel_viorel.frimu@stud.fils.upb.ro; 3 National Research and Development Institute for Gas Turbines COMOTI, 061126 Bucharest, Romania; alexandru.hank@comoti.ro

**Keywords:** teleoperation, hand tracking, human–robot interaction, vision-based control, robotic manipulation

## Abstract

Interactive teleoperation offers an intuitive pathway for human–robot interaction, yet many existing systems rely on dedicated sensors or wearable devices, limiting accessibility and scalability. This paper presents a vision-based teleoperation framework that enables real-time control of an articulated robotic arm (five joints plus a gripper actuator) using human hand tracking from a single, typical laptop camera. Hand pose and gesture information are extracted using a real-time landmark estimation pipeline, and a set of compact kinematic descriptors—palm position, apparent hand scale, wrist rotation, hand pitch, and pinch gesture—are mapped to robotic joint commands through a calibration-based control strategy. Commands are transmitted over a lightweight network interface to an embedded controller that executes synchronized servo actuation. To enhance stability and usability, temporal smoothing and rate-limited updates are employed to mitigate jitter while preserving responsiveness. In a human-in-the-loop evaluation with 42 participants, the system achieved an 88% success rate (37/42), with a completion time of 53.48 ± 18.51 s, a placement error of 6.73 ± 3.11 cm for successful trials (n = 37), and an ease-of-use score of 2.67 ± 1.20 on a 1–5 scale. Results indicate that the proposed approach enables feasible interactive teleoperation without specialized hardware, supporting its potential as a low-cost platform for robotic manipulation, education, and rapid prototyping.

## 1. Introduction

Natural and intuitive interaction modalities are becoming increasingly important in human–robot interaction (HRI), particularly for manipulation tasks where operator intent must be expressed quickly and reliably. Gesture-based interfaces have emerged as a practical pathway for touchless control, reducing dependence on conventional peripherals and enabling more accessible operation in training, laboratory, and collaborative settings [1,2].

Teleoperation remains a core solution when full autonomy is impractical or when safety, uncertainty, or task variability requires a human in the loop. In such systems, performance is often governed by the combined effects of perception robustness, command mapping, and end-to-end latency, which directly influence operator confidence and task success [3,4]. Modern teleoperation research spans high-speed visual teleoperation of dexterous end-effectors [3], whole-body or multi-DOF anthropomorphic control strategies [5], and grasp-oriented teleoperation methods emphasizing compliance and safety [6,7].

Recent advances in real-time hand tracking have made camera-only control increasingly feasible on consumer hardware, shifting part of the teleoperation stack from specialized sensors (e.g., gloves, inertial measurement units (IMUs), depth rigs) toward vision-based landmark pipelines and lightweight gesture descriptors. In the literature, hand-gesture interfaces have been used for robot path definition in collaborative workspaces [8]. Camera-only landmark pipelines and lightweight descriptor-based approaches have also been widely adopted to support practical gesture-driven interaction on consumer hardware [9,10,11], for telemanipulation based on hand motion interpretation and state estimation using wearable IMU sensors [12], and for gesture-driven robot programming approaches explicitly leveraging MediaPipe-style hand landmark extraction [13].

Despite this progress, mapping human hand motion to an articulated robotic arm remains nontrivial. Robots and human hands differ in kinematics, joint limits, and workspace constraints, while vision-based tracking introduces jitter, occlusions, and sensitivity to lighting and camera placement. These factors can degrade stability, increase perceived delay, and amplify user-to-user variability, which motivates control strategies that balance responsiveness with filtering and update constraints [14,15,16,17].

Recent studies also highlight a design continuum between (i) contactless vision interfaces and (ii) wearable or multimodal sensing solutions that increase precision at the cost of hardware complexity. For example, industrial robot control using gestures and voice has been demonstrated with depth-based sensing and real-time software integration [1], while other work analyzes the precision and stability limits of optical hand tracking that can impact control fidelity [2]. Wearable interfaces (e.g., IMU-based gesture control) remain relevant for assistive technologies where robustness is critical [18], and glove-based solutions continue to be used for teleoperated grasping and mapping stability improvements [19].

Recent teleoperation interfaces can be grouped into three main directions. First, vision-based approaches infer hand pose or gestures from RGB (or RGB-D) sensing and map them to robot commands, enabling uninstrumented interaction with commodity cameras and lightweight landmark pipelines [3,9,10,11,13,20,21]. Second, wearable or body-mounted sensing—such as data gloves, IMUs, or myoelectric armbands—can improve robustness and provide richer motion cues, but typically introduces additional hardware, calibration steps, and user instrumentation [6,7,18,19]. Third, in industrial/collaborative contexts, discrete gesture/voice interfaces are often used for high-level command selection or path definition, emphasizing interpretability and safety over continuous dexterity [1,8]. Across these directions, a recurring trade-off emerges between accessibility (minimal sensing and setup) and precision/robustness (richer sensing and modeling), motivating low-cost camera-only pipelines that remain usable under the occlusion, jitter, and depth ambiguity inherent to monocular hand tracking.

Despite these advances, mapping uninstrumented human hand motion to an articulated robotic arm remains nontrivial. Human hands and robotic arms differ in kinematics, joint limits, and workspace constraints, while monocular vision-based tracking introduces jitter, intermittent landmark loss, and depth ambiguity that can degrade stability and amplify user-to-user variability [2,14,21]. Prior work often emphasizes either discrete gesture interfaces or higher-fidelity sensing (e.g., wearables and sensor fusion) to increase robustness, whereas fewer studies report an end-to-end camera-only pipeline on consumer hardware together with a participant-level manipulation evaluation on a low-cost physical arm under a standardized protocol. This work targets that gap by combining a compact descriptor-to-joint mapping with stabilization/update policies and a controlled human-in-the-loop evaluation that reports success, time, attempts, placement error, and perceived ease of use.

In this paper, we present an interactive teleoperation system for an articulated robotic arm controlled using vision-based human hand tracking from a typical laptop camera. The pipeline extracts hand landmarks in real time, computes compact kinematic descriptors, maps them to joint-level servo commands using a calibration-based strategy, and transmits commands over a lightweight network interface to an embedded controller for actuation. The system is evaluated with 42 participants, reporting participant-wise task outcomes and timing, target accuracy, and a 5-point ease-of-use score, enabling mean and standard deviation reporting consistent with common evaluation practices.

Accordingly, the objectives of this study are: (1) to design an accessible, camera-only teleoperation pipeline that maps compact hand kinematic descriptors to joint-level commands for a low-cost articulated arm; (2) to implement a reproducible two-module software architecture (operator-side graphical user interface (GUI) plus embedded actuation server) with network-based command execution; (3) to evaluate the system in a structured human-in-the-loop study using a standardized pick-and-place task; and (4) to report participant-level outcomes (including failures and re-attempts) together with descriptive statistics to characterize usability and performance under first-exposure operation.

## 2. Materials and Methods

**Figure 1:**
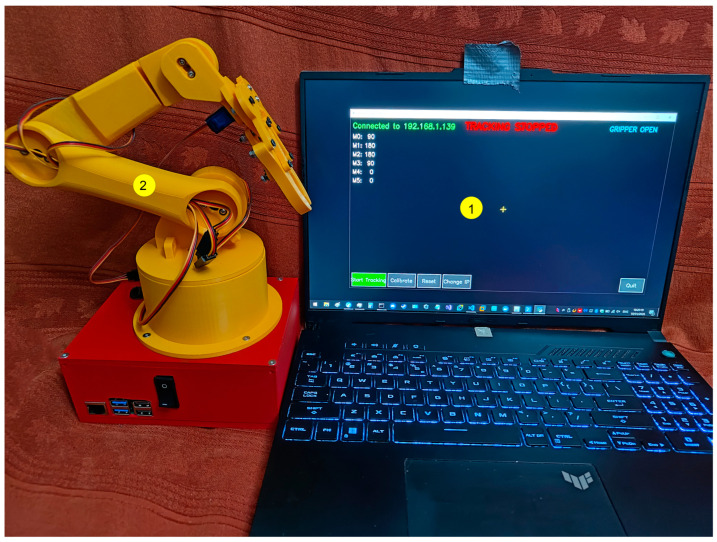
System overview of the interactive teleoperation setup: 1. operator-side GUI for vision-based hand tracking and command generation. 2. articulated robotic arm platform executing the received commands.

Figure 1 provides an overview of the proposed interactive teleoperation system, highlighting both the physical robotic platform and the operator-side software interface. The setup follows a camera-only, vision-based control paradigm: a laptop camera captures the user’s hand, a real-time hand-tracking pipeline estimates the hand landmarks, and a mapping layer converts compact hand descriptors into joint-level commands for an articulated robotic arm. These commands are transmitted over a lightweight network interface to an embedded controller that drives the servo motors through a PWM driver, enabling continuous low-cost teleoperation without wearable sensors or dedicated tracking hardware. The GUI supports the operational workflow by providing connection status, tracking state, calibration/reset controls, and a live visualization of the detected hand landmarks, which helps the operator maintain consistent hand pose and system feedback during teleoperation.

**Figure 2:**
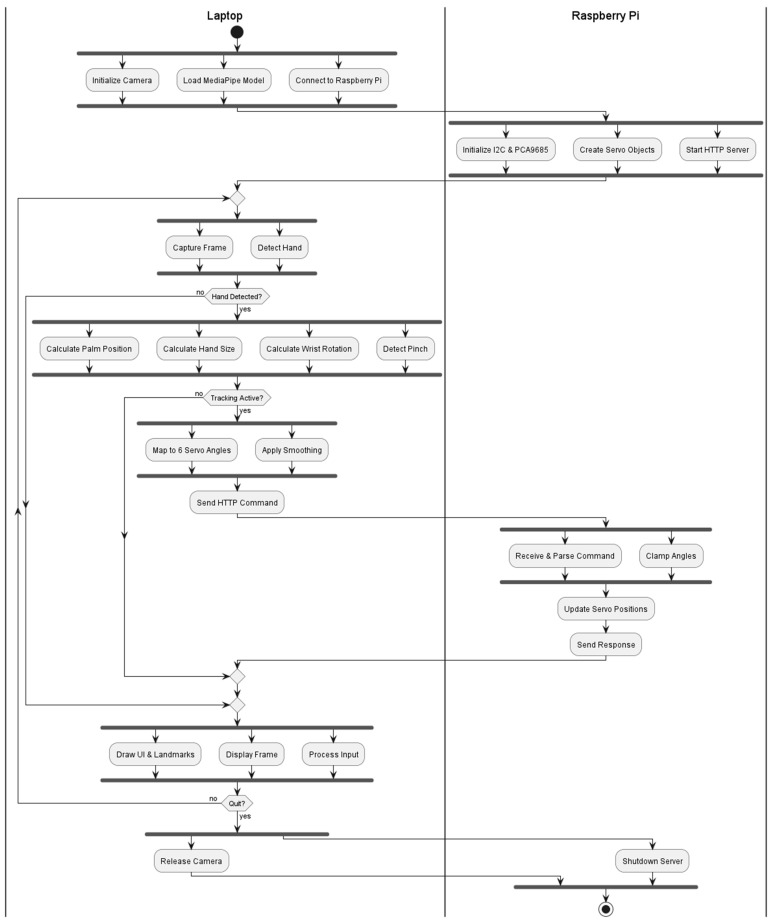
The activity diagram of the proposed system.

In Figure 1, the teleoperation setup is illustrated, including the articulated robotic arm and the operator-side graphical user interface used for vision-based hand tracking and command generation. The activity diagram of the proposed system is shown in Figure 2.

Figure 2 summarizes the end-to-end workflow of the proposed teleoperation system. Starting from monocular camera acquisition, the pipeline performs real-time hand landmark estimation, computes compact kinematic descriptors, applies stabilization (temporal smoothing and rate-limited updates), and transmits commands over the network to the embedded actuation server for PWM-based servo execution. Further implementation details for each stage are provided in Section 2.4.1.

### 2.1. Material Used for Printing

**Figure 3:**
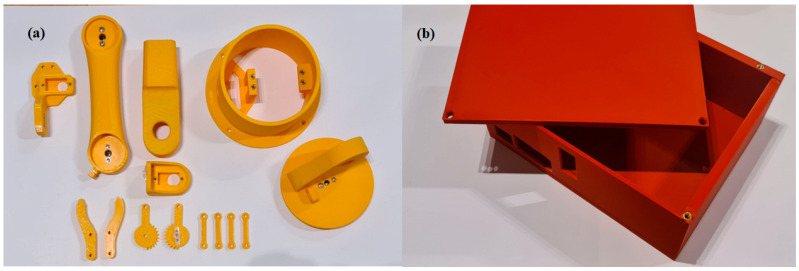
3D printed components: (**a**) Articulated arm robot’s segments. (**b**) Controller case.

All custom mechanical parts of the articulated arm and the controller enclosure were fabricated by fused deposition modeling (FDM) using polylactic acid (PLA) filament, which is a widely adopted, general-purpose thermoplastic for rapid prototyping and educational robotics. The 3D-printed arm segments used in the robotic structure are shown in Figure 3a, while the 3D-printed controller case housing the embedded electronics is illustrated in Figure 3b.

All custom components were fabricated by fused deposition modeling (FDM) using PLA filament. For reproducibility, the printer model and the main slicing/printing parameters used for all PLA parts are summarized in Table 1.

### 2.2. Technology Used

The proposed teleoperation system was developed using a lightweight software stack to enable interactive operation on consumer hardware. The operator-side module was implemented in Python 3.9.12, leveraging OpenCV for video capture and visualization and MediaPipe Hands for real-time hand landmark tracking from a monocular RGB stream.

The operator-side application was tested on a consumer laptop equipped with a Ryzen 7 7735HS, 16 GB of DDR5 RAM, and a Radeon 680M, running Windows 11 Version 25H2. The built-in webcam was used at a resolution of 1920 × 1080 and 30 fps capture settings. In practice, the pipeline requires a typical modern laptop capable of sustaining real-time webcam capture and landmark inference; the above configuration represents the tested reference platform for reproducibility.

The embedded actuation module runs on a Raspberry Pi and executes received motion commands to drive the robotic arm.

Communication between the operator module and the embedded controller is performed over a local network using a simple request–response interface, enabling modular deployment and straightforward reproducibility. Actuation is achieved through an I2C-based PWM driver to control multiple servos used by the articulated arm.

### 2.3. Hardware/Electronics Setup

The embedded actuation subsystem is built around a Raspberry Pi 5 (8 GB), which serves as the main controller and network endpoint for the teleoperation commands, booting from a 64 GB microSD card.

Real-time multi-servo control is achieved using a PCA9685 16-channel PWM driver connected to the Raspberry Pi via the I2C bus (SCL/SDA), enabling stable PWM generation for all joints. In the current prototype, six servo channels (0–5) are used to actuate the articulated arm and gripper, with high-torque MG996R servos employed for the main joints and a compact SG90 servo used where lower torque is sufficient (e.g., small joint/end-effector actuation). The PWM frequency is set to 50 Hz, which is the standard refresh rate for hobby servos.

Power is provided by a 2S 7.4 V 2200 mAh LiPo battery, with voltage regulation handled by an LM2596HVS step-down module to supply stable low-voltage rails for the control electronics and servo driver.

The system includes a panel-mounted power switch for manual power isolation and a 12 V cooling fan (powered at 7.4 V) for thermal management inside the controller enclosure during extended operation.

**Figure 4:**
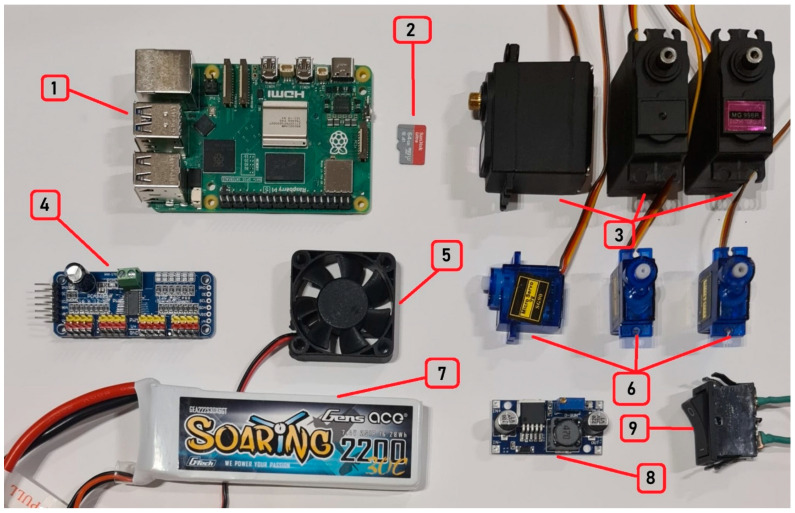
Articulated arm robot’s components: 1. Raspberry Pi 5 8 GB. 2. SanDisk 64 GB MicroSD Card. 3. MG996R Servo Motors. 4. PCA9685 Servo Driver. 5. 40 × 40 mm fan (12 V rated, powered at 7.4 V). 6. SG90 Servo Motors. 7. 2S 7.4 V 2200 mAh LiPo Battery. 8. LM2596HVS Step-Down Module. 9. Power Switch.

**Figure 5:**
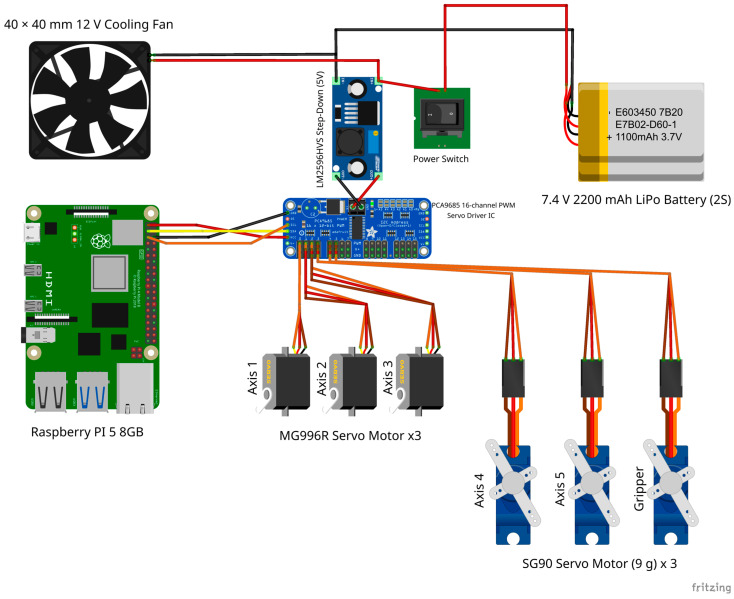
Circuit diagram made with Fritzing [22].

The 3D-printed controller case is shown in Figure 3b. The main embedded hardware components used in the actuation subsystem are illustrated in Figure 4, while the wiring diagram of the embedded control hardware and power distribution (Raspberry Pi–PCA9685–servo interconnect) is provided in Figure 5.

The robot platform comprises five revolute joints (5R) forming the arm kinematic chain, while a sixth actuator channel controls the gripper (open/close). In this 5R chain, the joints correspond to base rotation (J1), shoulder (J2), elbow (J3), wrist pitch (J4), and wrist rotation (J5), while the gripper is actuated separately.

### 2.4. Methods

#### 2.4.1. System Workflow and Operating Modes

The proposed teleoperation pipeline follows the activity flow shown in Figure 2 and is implemented as two cooperating software modules: (i) an operator-side application running on the laptop and (ii) an embedded actuation server running on a Raspberry Pi.

**Figure 6:**
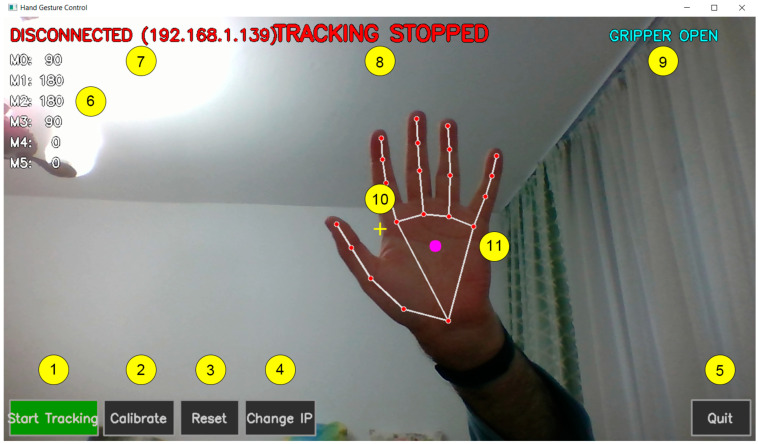
Application GUI: 1. Start/stop tracking. 2. Change/calibrate the point we use for tracking. 3. Reset arm position. 4. Change IP of the Raspberry Pi that is used to control the arm. 5. Exit the program. 6. Shows motor angles. 7. Shows connection status to the Raspberry Pi. 8. Shows current tracking status. 9. Shows gripper status. 10. Tracking center. 11. Hand landmark overlay.

An example of the operator-side interface, including the live hand landmark overlay and the on-screen system status indicators, is shown in Figure 6.

The gripper indicator reflects whether the gripper is currently open or closed, and the tracking indicator shows whether hand tracking is stopped or running. The tracking center marker represents the reference point used by the control algorithm to compute hand motion and generate the corresponding robot commands.

The operator-side GUI supports the experimental workflow by exposing start/stop tracking, calibration, reset, IP update, and live feedback (connection status, tracking state, and current servo angles). The embedded module exposes a minimal network interface with two endpoints—/status for connection/servo state queries and/command for command execution—allowing teleoperation to be started only after a valid link is confirmed.

#### 2.4.2. Operator-Side Hand Tracking and Feature Extraction

On the laptop, frames are acquired from the built-in webcam using OpenCV and processed in real time using MediaPipe Hands to estimate hand landmarks in a monocular RGB stream. Hand pose estimation (landmark detection and tracking) is performed using the off-the-shelf MediaPipe Hands pipeline, which provides real-time hand landmarks from monocular RGB frames. In this work, we do not modify the underlying landmark estimator; instead, we use its output as input to our control layer, which computes compact kinematic descriptors and maps them to robot actuation commands. The hand tracking subsystem uses the MediaPipe Hands model with static_image_mode = False (video-stream processing), max_num_hands = 1 (single-hand control), min_detection_confidence = 0.5 (initial hand detection), and min_tracking_confidence = 0.3 (temporal tracking continuity under challenging frames). These settings reflect the intended interactive teleoperation use case and were kept fixed across all experimental sessions. From the detected landmarks, the operator module computes compact kinematic descriptors that can be robustly evaluated at runtime: (i) palm position in the image plane, (ii) an apparent hand-size proxy (based on inter-landmark distance) used as a depth-related cue, (iii) wrist rotation (derived from the index MCP–pinky MCP axis), (iv) hand pitch (based on wrist-to-middle-finger geometry), and (v) pinch distance between thumb and index fingertip for gripper actuation. These descriptors are computed with lightweight NumPy operations, enabling continuous updates without requiring additional sensors or depth instrumentation.

#### 2.4.3. Calibration and Mapping to Joint Commands

A calibration step is performed to personalize the mapping and improve repeatability across users. When calibration is triggered, the system stores the current hand pose as reference (center palm location, reference hand size, reference wrist rotation, and reference hand pitch) and resets the arm to the default home configuration. During teleoperation, each descriptor is converted into a target joint angle for six servo channels (0–5). The mapping is designed to preserve intuitive correspondence between hand motion and robot motion: horizontal palm displacement drives base rotation (servo 0), vertical palm displacement drives arm tilt (servo 1), changes in apparent hand size drive arm extension/height behavior (servo 2), wrist rotation drives end-effector rotation (servo 3), hand pitch drives end-effector pitch (servo 4), and pinch state toggles the gripper between open and closed (servo 5). Servo commands are constrained to admissible ranges. On the operator side, all channels are bound to a nominal 0–180° interval, while the embedded controller additionally clamps the gripper channel to a reduced range (0–140°) to protect the mechanism.

An alternative would be task-space end-effector control using inverse kinematics (IK). In this work, we deliberately adopt a descriptor-to-joint mapping to minimize modeling overhead and dependence on precise kinematic calibration for a low-cost arm, and to maintain stable behavior under monocular depth ambiguity. Incorporating end-effector control with IK and constraint handling is a natural extension of the proposed framework.

Our contribution lies in the calibration-based descriptor-to-actuation mapping (including bounds and stabilization), rather than in training a new vision model for hand pose estimation.

#### 2.4.4. Command Stabilization and Update Policy

Because camera-only tracking can introduce frame-to-frame jitter, the operator module applies simple deterministic stabilization before transmitting commands. First, each servo target is filtered using a short temporal window implemented as a fixed-length deque buffer (three samples), and the filtered command is computed as the mean of the buffered values. Second, updates are rate-limited by enforcing a minimum inter-command interval of 0.03 s, preventing excessive network traffic and micro-oscillations during fast hand motion. Third, an update threshold is used such that a new command is transmitted only if the filtered angle differs by at least 2° from the last applied value, which reduces unnecessary corrections while preserving responsiveness. Gripper state is controlled using a pinch detector: if the thumb–index distance falls below a fixed threshold, the gripper is commanded closed; otherwise, it is commanded open.

#### 2.4.5. Network Interface and Embedded Actuation

Teleoperation commands are exchanged over a local network using an HTTP-based JSON protocol implemented with Python’s standard urllib library on the operator side. The Raspberry Pi runs a minimal HTTP server built on HTTPServer/BaseHTTPRequestHandler, exposing/status and/command endpoints. Servo actuation is performed through an I2C-controlled PWM driver (PCA9685) using Adafruit’s adafruit_pca9685 and adafruit_motor.servo libraries, configured at 50 Hz (standard hobby-servo refresh rate). To ensure safe concurrent access when commands arrive rapidly, servo state updates are protected by a lock, and the embedded software provides a gradual reset-to-home routine that steps the servos back to the default pose.

#### 2.4.6. Human-in-the-Loop Evaluation Protocol and Recorded Measures

The system is evaluated in a structured human-in-the-loop study with 42 participants. Participants self-identified as students on the informed consent form. To preserve anonymity and minimize personal data collection, no demographic attributes (e.g., age, gender, handedness, or prior robotics/teleoperation experience) were recorded or stored in the study dataset; participant outcomes were analyzed using anonymized identifiers.

The task is a standardized pick-and-place manipulation of a small rectangular box with dimensions 8.5 cm × 6.5 cm × 3.5 cm and a mass of 36 g. The workspace contains two marked areas on a flat surface: a start area where the object is initially placed and a target area where the object must be deposited. The center-to-center distance between the start area and the target area was 30 cm (measured on the tabletop). At the start of each session, the robot is reset to the home configuration and the object is placed in the start area with a consistent orientation. Each participant performs one familiarization trial (practice; not included in analysis) to understand the mapping and gripper gesture, followed by one measured trial. During the measured trial, the participant teleoperates the robot to approach the object, close the gripper to grasp it, lift it to clear the surface, transport it to the target area, and release it by opening the gripper. If the grasp fails or the object is dropped, the participant may reattempt within the same measured trial, with up to three attempts allowed; each re-grasp/retry is counted as an additional attempt.

For each participant’s measured trial, the following measures are recorded for subsequent statistical analysis: (i) task success/failure (successful if the object is released within the target area); (ii) completion time measured from trial start to task termination: for successful trials, from trial start to object release within the target area; for unsuccessful trials, from trial start to the end of the third allowed attempt (i.e., when the final failed grasp/drop occurs), since no valid target-area release is achieved; (iii) number of attempts executed within the measured trial (max. three); (iv) placement accuracy quantified as the planar distance (cm) between the center of the target area and the final resting position of the object after release (measured with a ruler); and (v) a single ease-of-use score reported on a 1–5 scale immediately after the trial. Ease-of-use was rated on a 1–5 Likert scale, where 1 = very difficult and 5 = very easy. Group-level summaries are reported across the 42 participants for all measures except placement error, which is computed over successful trials only (n = 37).

## 3. Results

The proposed vision-based teleoperation system was evaluated with 42 participants, each completing one familiarization trial (not included in the analysis) followed by one measured pick-and-place trial. The evaluation focused on task-level performance and usability outcomes, namely task success, completion time, number of attempts, placement accuracy, and ease-of-use (Table 2).

Across all participants, the system achieved a task success rate of 88% (37/42 successful trials). Considering all measured trials (including unsuccessful ones), the mean completion time was 53.48 ± 18.51 s, and participants required 2.24 ± 0.82 attempts on average. Subjectively, participants reported an average ease-of-use score of 2.67 ± 1.20 on a 1–5 scale. Placement accuracy (planar distance between the target center and the object’s resting position after release) was computed only for successful trials (n = 37), resulting in a mean placement error of 6.73 ± 3.11 cm.

**Figure 7:**
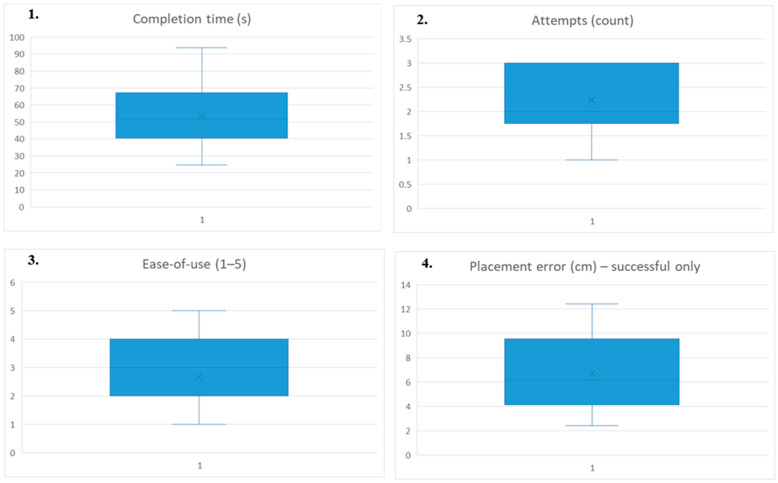
Distribution of participant-level outcomes in the measured trial (n = 42): (**1**) completion time (s). (**2**) number of attempts. (**3**) ease-of-use rating (1–5). (**4**) placement error (cm) computed for successful trials only (n = 37).

To complement Table 2, Figure 7 visualizes the distributions of completion time, number of attempts, ease-of-use ratings, and placement error (successful trials only, n = 37). These plots provide a compact view of dispersion and outliers across participants.

Descriptive statistics are reported as mean ± sample standard deviation. For the binary success outcome, the primary summary is the success rate (successful trials/total), while placement error statistics are computed for successful trials only (n = 37).

The results also indicate noticeable variability across participants. For successful trials, completion time ranged from 24.72 s to 74.01 s; when unsuccessful trials are included, completion time extended up to 93.75 s. Placement error ranged from 2.4 cm to 12.4 cm, suggesting differences in fine positioning and release control during teleoperation. The number of attempts varied between 1 and 3, reflecting the frequency of re-grasp events and recovery actions during the measured trial. Ease-of-use scores ranged from 1 to 5, indicating user-to-user differences in perceived intuitiveness and control stability under camera-only hand tracking. These outcomes provide context for the discussion of teleoperation mapping, tracking jitter, and the stabilization/update policy in the subsequent section.

## 4. Discussion

The results demonstrate that a camera-only, vision-based interface can support interactive teleoperation of an articulated robotic arm without dedicated sensing hardware. In the human-in-the-loop study (n = 42), the system achieved an overall success rate of 88% (37/42), with a mean completion time of 53.48 ± 18.51 s and 2.24 ± 0.82 attempts per participant. Placement accuracy, evaluated only for successful trials (n = 37), yielded a mean planar error of 6.73 ± 3.11 cm, while the ease-of-use score (1–5) showed substantial dispersion (2.67 ± 1.20), indicating non-negligible user-to-user variability. Taken together, these findings support the feasibility of low-cost interactive teleoperation based on monocular hand landmarks, while also highlighting operational limitations that emerge when mapping human hand motion to multi-joint actuation under real-world tracking noise.

A key observation is that performance variability is strongly influenced by the combined effects of (i) kinematic mismatch between a human hand and an articulated arm, and (ii) vision-driven measurement instability. The widest spread appears in completion time (24.72–93.75 s across all trials) and in the number of attempts (1–3), suggesting that failures and re-grasp events are primary contributors to time overhead. This aligns with known challenges in teleoperation where perception robustness, mapping sensitivity, and feedback delays interact to affect operator confidence and task execution [3,4]. In our setup, failures tended to coincide with the maximum number of allowed attempts and longer completion times, which is consistent with the idea that grasp acquisition and stable transport dominate the difficulty for novice users.

From a control-design standpoint, the stabilization and update policy used here (short temporal smoothing, a minimum inter-command interval, and a minimum angle-change threshold) represents a pragmatic trade-off: reducing frame-to-frame jitter at the cost of “micro” responsiveness. Such trade-offs are widely motivated in camera-only hand tracking because occlusions, lighting sensitivity, and landmark jitter can produce high-frequency command oscillations and unstable end-effector motion [14,15,16]. The present results suggest that the adopted policy is sufficient to enable successful manipulation for most users, but the observed dispersion in time and error indicates that stabilization alone does not fully address variability introduced by (i) inconsistent camera placement and hand visibility, (ii) monocular depth ambiguity (approximated via apparent hand scale), and (iii) differences in user strategy and prior familiarity with teleoperation.

When contextualized against related work, the contribution of this study is primarily its end-to-end, reproducible, camera-only teleoperation pipeline and its participant-wise evaluation. Prior studies have demonstrated gesture and multimodal command interfaces for robot control in industrial or collaborative contexts [1,8], as well as vision-based teleoperation for dexterous end-effectors where responsiveness and perception quality are critical [3]. Other approaches improve robustness through wearable sensing (e.g., IMUs, gloves, or myoelectric armbands), which can increase control precision but introduce additional hardware and calibration overhead [6,7,12,18,19]. By contrast, the present system deliberately targets accessibility—monocular RGB hand landmarks (MediaPipe-style) on consumer hardware [9,10,11]—and shows that, even with this minimal sensing, structured manipulation is achievable with a meaningful success rate and measurable task performance. Compared to depth-assisted landmark pipelines, which can improve 3D consistency and mitigate monocular ambiguity [21], our approach trades accuracy for simplicity, which is reflected in the placement error magnitude and the reliance on user compensation during fine positioning.

The ease-of-use results provide additional insight into usability and learning effects. The wide spread of ratings (1–5) and the mid-range mean suggest that the interface is not uniformly intuitive for first-time users, despite calibration and on-screen feedback. This is consistent with the broader literature showing that camera-based hand interfaces can be sensitive to individual differences in hand pose, motion style, and the ability to maintain stable visibility under a fixed camera viewpoint [2,14]. Operationally, users who naturally maintain a steady hand pose and avoid self-occlusion can more readily exploit the mapping, whereas users with larger motions, occlusions, or unstable depth cues may experience higher jitter and require more corrective actions, increasing attempts and time.

To contextualize our results quantitatively, Table 3 summarizes the key performance indicators reported in this work and in the main related teleoperation/control interfaces already discussed above (note that robots, tasks, and metric definitions differ across studies, so values should be interpreted as indicative).

### 4.1. Limitations

Several limitations should be acknowledged. First, placement error was measurable only for successful trials; thus, the reported accuracy reflects successful executions and does not capture spatial behavior in failures. Second, the protocol uses a single measured trial per participant (after one familiarization trial), which is appropriate for capturing first-exposure usability but does not quantify learning curves across repeated sessions. Third, the study focuses on a single lightweight object and a planar start–target transfer, which represents a constrained manipulation scenario; additional tasks (e.g., different object shapes, clutter, longer transport distances, or variable target sizes) may reveal further differences in control stability. Future work will systematically vary object size/material and ambient illumination levels, and will increase scene complexity (e.g., clutter), to better assess generalization. Fourth, the camera-only hand-tracking pipeline is sensitive to lighting changes, self-occlusions, and camera placement, which can introduce jitter and transient landmark loss that users must compensate for during fine positioning. Finally, the system uses position-based servo control without force/impedance feedback, which limits suitability for contact-rich manipulation; integrating compliance strategies or richer feedback modalities would be needed for higher-fidelity telemanipulation, as emphasized in related teleoperation research on grasping and safe interaction [5,6,7].

Because demographic and prior-experience variables were not recorded, stratified analysis (e.g., by handedness or prior familiarity) is not possible and may contribute to the observed inter-participant variability.

### 4.2. Future Work

Future work can strengthen both robustness and usability while preserving the low-cost goal. On the perception side, multi-view capture or improved occlusion handling could reduce sensitivity to camera placement [15], while depth-enabled landmark estimation could mitigate monocular depth ambiguity [21]. On the control side, user-adaptive motion scaling, per-joint deadbands tuned to noise statistics, and task-aware filtering (e.g., stronger smoothing during fine placement and weaker smoothing during gross motion) may reduce corrective oscillations while maintaining responsiveness. Finally, reporting additional objective measures (e.g., separate timing for grasp, transport, and placement, or jitter metrics in commanded angles) and introducing a simple baseline condition (e.g., keyboard/slider control or a wearable alternative) would allow more direct quantitative comparison with established teleoperation modalities.

The 5R and gripper configuration is sufficient for the standardized pick-and-place task considered here, but it does not target full hand-like dexterity; extending to higher-DOF manipulators is future work.

## 5. Conclusions

This paper presented an interactive, camera-only teleoperation system for an articulated robotic arm based on vision-driven human hand tracking. The proposed pipeline extracts hand landmarks from a standard laptop camera, computes compact kinematic descriptors, maps them to joint-level servo commands through a calibration-based strategy, and transmits commands over a lightweight network interface to an embedded controller for actuation. A lightweight stabilization policy (short temporal smoothing, update rate limiting, and minimum-change thresholds) was adopted to mitigate tracking jitter while maintaining interactive responsiveness.

In a human-in-the-loop evaluation with 42 participants, the system achieved an overall task success rate of 88% (37/42) in a standardized pick-and-place task. The mean completion time across all trials was 53.48 ± 18.51 s, with 2.24 ± 0.82 attempts, while placement accuracy computed for successful trials (n = 37) yielded a mean error of 6.73 ± 3.11 cm. Participants reported an ease-of-use score of 2.67 ± 1.20 (1–5), indicating substantial variability in perceived intuitiveness across first-time users.

Importantly, the dispersion in completion time and the number of attempts suggests that re-grasp events and failure recovery dominate the time overhead for first-time users under monocular ambiguity and intermittent landmark loss.

Together with the quantitative context introduced in Table 3, these outcomes position the proposed camera-only approach as a practical baseline for accessible teleoperation with participant-level performance reporting.

Overall, these results support the feasibility of low-cost, wearable-free teleoperation using monocular hand landmarks, with potential utility as a reproducible platform for education, rapid prototyping, and accessible HRI experimentation. Future work will focus on improving robustness to occlusions and viewpoint changes, enhancing mapping adaptivity (e.g., user-specific scaling and task-aware filtering), extending evaluation to more diverse manipulation tasks and objects, and introducing baseline comparisons against alternative control modalities.

## Figures and Tables

**Table 1:** FDM 3D-printing parameters used for all PLA components.

3D Printer	Material	Infill	Infill Type	Nozzle/Printing Peak Temperature	Print Speed	Layer Height	Support Type	Support Threshold Angle
Bambu Lab A1 Mini	PLA	15%	Gyroid	275 °C	150 mm/s	0.2 mm	Tree (auto)	60°

**Table 2:** Participant-level results for the measured pick-and-place trial (n = 42).

Participant	Success (0/1)	Completion Time (s)	Attempts (1–3)	Placement Error (cm)	Ease of Use (1–5)
1	1	42.13	2	3.4	2
2	0	91.23	3	-	5
3	1	24.72	1	6.2	3
4	1	62.84	3	7.3	4
5	1	34.15	3	10.1	3
6	1	45.23	2	6.4	2
7	1	52.07	3	3.2	3
8	1	37.79	1	2.4	1
9	1	48.21	3	11.3	4
10	0	82.18	3	-	4
11	1	25.14	1	8.4	3
12	1	52.62	2	5.8	2
13	1	47.18	3	3.2	2
14	1	51.82	2	4.3	3
15	1	46.23	2	3.1	3
16	1	52.11	1	4.4	2
17	0	87.91	3	-	5
18	1	70.22	3	11.1	4
19	1	46.31	2	12.3	2
20	1	51.43	1	9.7	1
21	1	27.45	3	3.1	3
22	1	63.12	3	10.4	4
23	1	49.88	3	4.2	2
24	1	31.50	2	12.1	1
25	1	74.01	1	12.4	3
26	0	85.09	3	-	5
27	0	93.75	3	-	4
28	1	58.23	2	3.0	2
29	1	25.99	3	9.4	2
30	1	66.75	3	7.5	3
31	1	41.30	3	5.3	1
32	1	38.05	3	6.2	4
33	1	52.19	2	8.6	2
34	1	70.64	1	4.0	1
35	1	29.47	3	7.1	3
36	1	61.00	2	5.3	3
37	1	55.82	2	9.4	4
38	1	34.16	3	4.2	2
39	1	72.33	1	10.3	1
40	1	44.91	2	5.5	2
41	1	50.55	1	3.2	1
42	1	68.27	1	5.1	1
Mean	0.88	53.48	2.24	6.73	2.67
SD	0.33	18.51	0.82	3.11	1.20

Note: SD = Standard Deviation; Placement error statistics were computed for successful trials only (n = 37).

**Table 3:** Quantitative benchmarking against the main related works already discussed in this section.

Work	Interface/Sensing	Evaluated Scenario (as Reported)	Evaluation Scale	Reported Quantitative Results
This work	Monocular RGB camera (MediaPipe-style landmarks), wearable-free	Pick-and-place with articulated arm (human-in-the-loop)	n = 42 participants	Success: 88% (37/42); Time: 53.48 ± 18.51 s; Planar placement error: 6.73 ± 3.11 cm (successful trials only)
[7]	Wearable myoelectric armband (sEMG) and FSM control	Pick-and-place tasks with gripper/dexterous hand (multiple viewpoints); closest to ours: Task 1 (Gripper, Front)	n = 15 participants	Task 1 time: 101.4 ± 12.6 s; Task 1 translational path: 1.87 ± 0.21 m (reported for the authors’ Hybrid_All method)
[8]	Gesture-based interface with RGB-D sensors and digital twin feedback	Robot path/waypoint definition in collaborative workspace	n = 20 volunteers	Mean time (gesture-based): 23.69 s (SE 0.49); Mean deviation: 0.0408 m (SE 0.0019) (compared vs. teach pendant/hand guiding in the same study)
[21]	Depth–MediaPipe and dynamic hand gestures (RealSense-type depth pipeline)	Complete “move–pick–place–drop” task (quadruped and mounted arm)	15 grasping experiments	Success: 86.67% (13/15); Avg. time: 57.69 s; Avg. recognition delay: 29.77 ms

## Data Availability

The original contributions presented in this study are included in the article. Further inquiries can be directed to the corresponding author.
